# IL33 and Mast Cells—The Key Regulators of Immune Responses in Gastrointestinal Cancers?

**DOI:** 10.3389/fimmu.2020.01389

**Published:** 2020-07-03

**Authors:** Moritz F. Eissmann, Michael Buchert, Matthias Ernst

**Affiliations:** Olivia Newton-John Cancer Research Institute, and La Trobe University School of Cancer Medicine, Heidelberg, VIC, Australia

**Keywords:** interleukin 33 (IL33), mast cell (MC), innate immunity, ST2, gastrointestinal (GI) malignancies, tumor microenvironment (TME), therapy targets, cytokine signaling

## Abstract

The Interleukin (IL-)1 family IL33 is best known for eliciting type 2 immune responses by stimulating mast cells (MCs), regulatory T-cells (Tregs), innate lymphoid cells (ILCs) and other immune cells. MCs and IL33 provide critical control of immunological and epithelial homeostasis in the gastrointestinal (GI) tract. Meanwhile, the role of MCs in solid malignancies appears tissue-specific with both pro and anti-tumorigenic activities. Likewise, IL33 signaling significantly shapes immune responses in the tumor microenvironment, but these effects remain often dichotomous when assessed in experimental models of cancer. Thus, the balance between tumor suppressing and tumor promoting activities of IL33 are highly context dependent, and most likely dictated by the mixture of cell types responding to IL33. Adding to this complexity is the promiscuous nature by which MCs respond to cytokines other than IL33 and release chemotactic factors that recruit immune cells into the tumor microenvironment. In this review, we integrate the outcomes of recent studies on the role of MCs and IL33 in cancer with our own observations in the GI tract. We propose a working model where the most abundant IL33 responsive immune cell type is likely to dictate an overall tumor-supporting or tumor suppressing outcome *in vivo*. We discuss how these opposing responses affect the therapeutic potential of targeting MC and IL33, and highlight the caveats and challenges facing our ability to effectively harness MCs and IL33 biology for anti-cancer immunotherapy.

## Introduction

The tumor microenvironment (TME) is a complex collection of cellular and extra cellular matrix (ECM) components. Interactions and communications between the various components of the TME are orchestrated by a multitude of signaling molecules, including the cytokine interleukin (IL)33. IL33 was first discovered in 2003 as a nuclear factor in HEVEC cells (NF-HEV) (1) and later identified as an IL1 family cytokine and ligand for the interleukin 1 receptor like 1 receptor (IL1RL1, or commonly referred to as ST2) (2).

IL33 is expressed in fibroblasts, endothelial and epithelial cells (1, 3, 4) as well as in many cancer cells [reviewed in (5, 6)]. Depending on stimulation or disease context, this cytokine is produced by additional cells such as MCs (7), dendritic cells, macrophages, neutrophils, eosinophils, B cells and red blood cells (8–11). Anatomically, the expression of IL33 is highest in barrier tissues like the skin, the air ways and the GI tract, where IL33 release activates innate and adaptive immune responses upon tissue injury or various infections [reviewed in (12)]. Indeed, tissue resident innate immune cells are the proposed first responder for released IL33, and MCs are present at all these environment-tissue interfaces (13). In general, necrotic or necroptotic cell death is required for its release (14–21), nevertheless, multiple studies suggest release of IL33 from living cells (22–25), suggesting various modes of active secretion and passive release with and without necrotic/necroptotic cell death depending on cell type and stimuli. Further research is required to unravel the exact mechanisms of IL33 release.

IL33 cytokine exerts its activity via binding to a heterodimeric receptor consisting of its primary receptor ST2 and a co-receptor, IL1 receptor accessory protein (IL1RAP) (26, 27) triggering downstream signaling pathways including MYD88, IRAK1/4, MAP kinases and NF-kB (2, 12). Importantly, the various biological outputs following engagement of the IL33-ST2 axis are heavily dictated by the cellular context, which we will further summarize in this review, with a special focus on interaction and importance the innate-immune mast cells for IL33 signaling in cancer. Besides acting as an extracellular ligand conferring activity through its cognate ST2 receptor on targets cells, ST2-independent nuclear IL33 can act as transcriptional repressor in fibroblast, endothelial and immune cells (28, 29). Likewise, nuclear IL33 also promotes immune suppressive functions independent of ST2 in regulatory T (Tregs) cells (30), and cell intrinsic IL33 plays a role in B cell development (31).

## IL33—Responsive Cells in the Tumor Microenvironment

Since the identification of ST2 as the cognate receptor of IL33, various cell types have been shown to express ST2 and to respond to IL33 stimulation. However, there is a significant difference in the quality and quantity of ST2 expression among various cell types. Innate lymphoid cell type 2 (ILC2), Tregs and MCs express the ST2-receptor constitutively, while all other cell types that respond to extracellular IL33 are either ST2 negative at steady-state and only induce ST2 expression upon activation, or express ST2 on minor cell subsets in specific biological processes in a tissue-dependent manner (32).

### ILC2 Cells

A significant subset of innate lymphoid cell type 2 (ILC2) are constitutive ST2 expressers. However, the proportion of ST2 positive ILC2s can vary depending on tissue origin and disease context (32–37). Stimulation of ILC2s by IL33/ST2-signaling is critical for their activation, induces their expansion within tissues and triggers secretion of the type 2 cytokines IL-5 and IL-13. This classic type 2 (innate) immune response contributes to anti-helminth immunity, lipid metabolism and to the development of various allergic diseases such as asthma, atopic dermatitis, allergic rhinitis, and chronic rhinosinusitis (12, 13, 38–40). Recently, it was reported that IL33-activated tumor infiltrating ILC2s (TILC2) restrict pancreatic tumor growth. Moreover, IL33 induces the expression of inhibitory checkpoint receptor PD-1 in TILC2s. Antibody-mediated PD-1 blockade leads to TILC2 expansion and activation, resulting in augmented anti-tumor immunity, and enhanced tumor control (41).

### Treg Cells

Depending on the tissue and disease setting, a significant proportion of Tregs constitutively express the ST2 receptor (32–37). IL-33/ST2 signaling in Tregs has been shown to promote Treg frequency and immunosuppressive capacity in colitis and tissue injury models as well as graft vs. host disease (35, 42). In cancer, IL33/ST2 signaling in Tregs seems particularly important in colon cancer, where the frequency of ST2-expressing Tregs is higher and ST2-expression is upregulated compared to normal colon tissue. Signaling through the ST2 receptor can increase frequency, activity and migratory potential of Tregs, which is associated with increased colonic tumor burden (43–45). However, there are also studies that demonstrate increased Treg density upon genetic ST2 ablation (34).

### Mast Cells

While MCs can confer their functions through cell-cell contacts, their predominant way of shaping their cellular environment occurs via release of preformed or newly synthesized mediators. These paracrine acting molecules include growth factors, proteases, leukotrienes, cytokines and chemokines which in turn modulate biological processes and responses including: tissue remodeling, angiogenesis, pro/anti-inflammatory responses, immunosuppression, and cellular proliferation, survival, recruitment, maturation and differentiation (46, 47).

MCs provide critical nodes for IL33 signaling in innate immune cells. In external surface organs, where epithelial cells express high levels of IL33, the number of MCs is highest (48). MC's are first responders during infections, where IL33 acts as an alarmin following its release as a cellular danger signals (49). The dual importance of IL33 and MCs in allergies is well established (50), yet critical roles for the IL33-MC axis have also been uncovered in allergic, autoimmune, inflammatory disease as well as cancer and other diseases (51, 52). In addition, MCs can potentiate the biological impact of IL33, because chymases and tryptases released by activated MCs process full-length IL33 into a truncated and biologically more active mature protein (53). In addition, MCs have been described to also produce IL33 (7).

**Figure 1:**
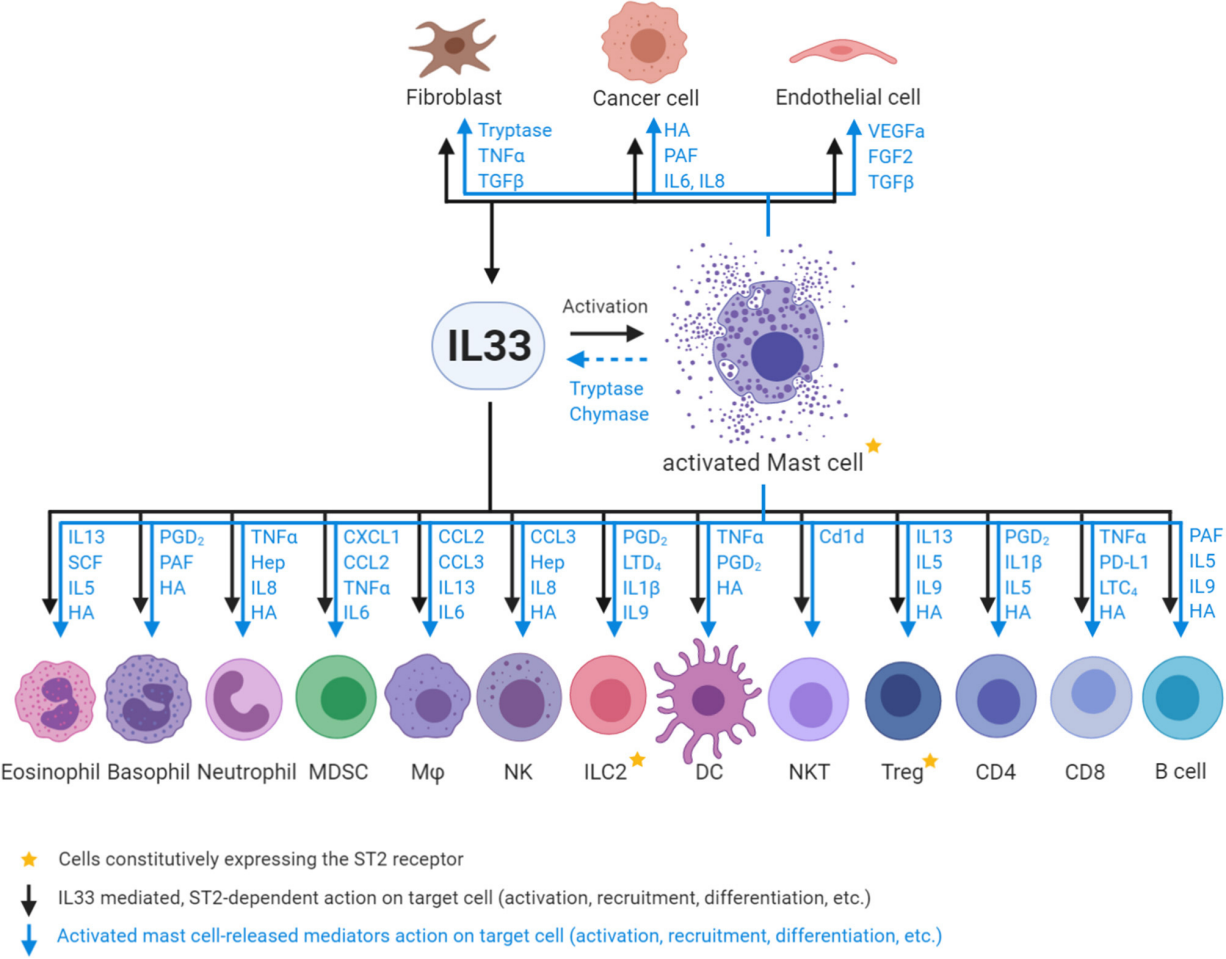
Interactions between IL33, activated MCs and ST2-positive responder cells. Fibroblasts, endothelial and epithelial/tumor cells are the major source of IL33 in the tumor microenvironment, and can in turn be stimulated by IL33. IL33 activates MCs and in turn MC -released chymases/tryptases cleave full length IL33 into highly active mature IL33. Subsequently, both IL33 (via ST2 receptor binding on target cells) and activated MC (via mediator release; depicted in blue) action innate immune cells: eosinophils, basophils, neutrophils, myeloid derived suppressor cells (MDSC), macrophages (Mϕ), natural killer cells (NK), type 2 innate lymphoid cells (ILC2), dendritic cells (DC), and adaptive immune cells: natural killer T cells (NKT), regulatory T cells (Treg), CD4 T cell subsets (Th1/2/17), CD8 T cells (CD8), and B cells. Mast cell mediator abbreviations: TNFα, Tumor necrosis factor alpha; TGFβ, Transforming growth factor beta; HA, Histamine; PAF, Platelet activating factor; IL, Interleukin; VEGFa, Vascular endothelial growth factor A; FGF2, Fibroblast growth factor 2; SCF, Stem cell factor; PGD_2_, Prostaglandin D_2_; Hep, Heparin; CXCL1, C-X-C-motif chemokine; CCL, C-C motif chemokine ligand; LTD_4_, Leukotriene D_4_; Cd1d, Cluster of differentiation 1 family glycoprotein; PD-L1, Programmed death-ligand 1; LTC_4_, Leukotriene C_4_.

MCs appear to be the only cell type which constitutively express high levels of ST2 independent of their tissue origin or maturation/activation status (33, 54). Importantly, activation of MCs by IL33 leads to the release of a plethora of factors that act on various cell types in the TME and influence their recruitment, rate of proliferation and their state of activation, differentiation and polarization (Figure 1) (46, 55–65).

The striking overlap of cell types which respond to IL33 and mast cell-released mediators highlights the importance of the IL33-MC axis for the biological outcome and demonstrates the potential of MCs as amplifiers and regulators of IL33-mediated processes. However, most past studies have investigated the roles of IL33/ST2 and of MCs separately. We and others have begun to better integrate these closely related aspects of innate cell biology in the context of GI cancer, since this organ system is known for both high IL33 expression and high density of MCs.

### Other Cell Types

Besides the constitutively ST2-expressing ILC2, Tregs and MCs, there are various cell types, which don't express ST2 at steady state but expression can be induced or is present in minor cellular subsets. These include endothelial cells (66, 67), epithelial and epithelial-derived cancer cells (68, 69), fibroblasts (34, 70) and other non-immune cell types. Importantly, fibroblasts, endothelial and epithelial cells are also the major cellular sources of IL33 production in the tumor microenvironment (Figure 1) (3–5). The immune cells that respond to IL33 in a ST2-dependent manner (in addition to MCs, Tregs and ILC2s) are the innate immune cells: eosinophils, basophils, neutrophils, myeloid derived suppressor cells (MDSC), macrophages (Mϕ), natural killer cells (NK), dendritic cells (DC), and the adaptive immune cells: natural killer T cells (NKT), CD4 T cell subsets (Th1/2/17), CD8 T cells, and B cells (Figure 1) (71–80).

## IL33 and Mast Cells in Gastrointestinal Cancer

Various reviews try to group the IL33-responding immune cell types based on their role in tumor growth, whereby MCs, (tumor associated) macrophages and Tregs are considered pro-tumorigenic, while CD8, NK, NKT, and DC conferring predominantly anti-tumorigenic functions (6, 74, 77, 81). Beside these “classical activities,” for many of these cells both anti- and pro-tumorigenic roles have been described and for most cell types their functions might be tumor-type and -stage dependent.

The role of IL33 in cancer has been reviewed recently (81, 82). IL33 expression correlates with poor prognosis in some cancers, but predicts good outcomes in others (77). Likewise for MCs, high mast cell infiltration can correlate either with poor or good prognosis depending on the tumor type (65).

### Pre-cancerous Inflammation

Chronic inflammation or infection often precedes neoplastic transformation. Accordingly, IL33 expression is elevated in colonic epithelial cells and myofibroblasts of ulcerative colitis patients (83, 84) and in the chronically inflamed stomachs of patients infected with *H. pylori* or during bouts of acute gastritis (85, 86). Meanwhile, increased MC numbers are readily detected in patients with ulcerative colitis, gastritis and various other inflammatory disorders of the GI tract [reviewed in (87)] and have been attributed a disease-promoting role (88).

Conversely, simultaneous ablation of MCP-6/7, mouse orthologs of the human b tryptases TSAB1/2, significantly protected mice from dextran sodium sulfate (DSS)-induced colitis (89). While thi observation suggests that MCs may promote the inflammatory environment that mediates DSS-dependent destruction of the epithelial layer, the role of MC during the subsequent “wound-healing reaction” remains less clear. Although, it has been noted that tryptase-expressing MCs persist for several weeks at the site of the original injury (90). Consistent with a role for MC to not only release various leukocyte attracting chemokines, but to also induce proliferative effects on fibroblasts and other “bystander” cells (91). In turn, soluble factors from fibroblasts, including IL-33 can then feed-forward on MC and shape their phenotype (92). Indeed, in response to DSS administration, IL33 activated MCs in the colonic epithelium, which subsequently promoted restoration of epithelial barrier function and regeneration of epithelial tissues (93). In accordance with this, Rigoni et al. observed exacerbated colitis in MC-deficient Kit^w−Sh^ mice (94). Collectively these preclinical studies suggest a functional connection between IL33 and MCs during inflammation-associated regeneration of the GI epithelium. Similarly, tumors, “wounds that do not heal,” may co-opt these wound-healing associated IL33-mast cell immune responses (95).

### Intestinal and Colorectal Cancer

Although IL33 is elevated in colorectal cancer (CRC) patients when compared to normal tissues, in some studies its levels were reduced when comparing late vs. early stage disease (70, 96–98). Mast cell infiltration is associated with poor prognosis in colorectal cancer patients [reviewed in (65)], and at least one study also associated high IL33 expression with poor survival outcomes for metastatic CRC (99). Meanwhile, IL33-ST2 mechanisms underpinning pro- and anti-tumoral roles in CRC have been studied in mice. Maywald et al., observed reduced intestinal polyposis in IL33-deficient Apc^Min^ mice, which was associated with a lack of IL33-mediated mast cell and myofibroblast activation (70). A tumor promoting role for IL33 was confirmed independently (44). However, two separate studies reported elevated tumor burden in MC-deficient Apc^Min^ mice when compared to their MC-proficient counterparts (100, 101). Meanwhile, intestinal polyps in Apc^Δ468^ mutant mice have increased IL33 expression and reduced numbers of MCs contribute to the anti-tumoral effect of IL10-deficiency (54) and 5-lipoxygenase-deficiency (102).

In the classic carcinogen-induced mouse model of sporadic colon cancer (6x AOM), colon tumors displayed increased expression of IL33 and ST2. However, mast cell numbers were unchanged, while ST2-deficieny increased number and size of the colon tumors. Surprisingly, the tumor suppressive role of the IL33-ST2 signaling pathway occurred independently of MC abundance, but was mediated by mesenchymal (stem) cells and associated with a strong interferon gamma (IFNγ) gene expression signature (34).

However, in the AOM/DSS inflammation-associated CRC model, ST2-deficient mice had reduced tumor burden, possibly owing to ST2-expressing Tregs although these authors neither investigated the number nor activation status of MCs (43). Using the same model, Mertz et al. also observed reduced tumor burden in ST2-deficient mice (98). Using adoptive bone marrow chimeras, these authors attributed the anti-tumor effect to both the radio-resistant and radio-sensitive cell compartments and demonstrated an involvement of several hematological cell types (98). The latter observation was consistent with earlier work demonstrating reduced colonic tumor burden in MC-deficient c-Kit^W−sh^ mice following the AOM/DSS challenge (94).

### Gastric Cancer

IL33-mediated spasmolytic polypeptide-expressing metaplasia (SPEM) in the stomach of mice is associated with a strong Th2 cytokine response, suggesting an involvement of MCs (103). In human gastric cancer cell lines, IL33 promoted epithelial-to-mesenchymal transition *in vitro* and xenograft tumor growth in an ST2-dependent manner (104). Recently, we illustrated that MC numbers are elevated in human gastric cancer specimens and that high expression of an IL33-MC activation gene signature predicts poor survival of intestinal-type gastric cancer in patients (33). Utilizing mouse models, we identified an IL33-MC-macrophage axis promoting gastric cancer growth where either ST2-deficiency, lack of MCs or lack of macrophages all restricted gastric cancer growth in the preclinical *gp130*^*FF*^ mouse model of inflammation-associated gastric cancer. IL33-mediated activation of MCs and subsequent secretion of macrophage attracting factors form part of a mechanism resulting in the accumulation of pro-angiogenic and pro-tumorigenic macrophages in the gastric tumors. In ST2-deficient *gp130*^*FF*^ mice, ILC2 and Treg density was not altered, while frequency of MCs was decreased and associated with reduced tumor growth. Conversely, adoptive transfer of ST2-proficient MC stimulated tumor growth in ST2-deficient *gp130*^*FF*^ mice, demonstrating that IL33-ST2 signaling within MCs is part of the tumor promoting effect of IL33 in gastric cancer (33).

### Other Cancers of the Gastrointestinal Tract

IL33 administration promoted the growth of Kras and TGFbR2 mutant biliary tract cancers (105) and in mouse models with constitutively active AKT/YAP pathway (106, 107). Moreover, IL33 is overexpressed in human gallbladder cancer patients (108). However, in pancreatic cancer patients high IL33 expression and high number of tumor-infiltrating ILC2s correlated with better survival (41). This is consistent with the observation in a pancreatic cancer mouse model, that IL33 activated tumor–associated ILC2s mediated anti-tumor immunity. MCs were not investigated in this study, even though MC's pancreatic tumor promoting functions are known (109). Finally, IL33 is highly expressed in patients with esophageal squamous cell carcinoma. In corresponding cell lines, IL33 overexpression promoted migration and invasiveness, while IL33 knockdown inhibited the metastatic potential of these cells (110).

## Therapeutic Targeting of the IL33-MC Axis

In recent years, a number of studies have identified compounds that inhibit IL-33 mediated activation of MCs. Amongst those are natural compounds from plants like berberine (111), methoxyluteolin (112), and resveratrol (113) or ES-62 produced from parasitic worms (114) as well as various other drug classes including didox (synthetic ribonucleotide reductase inhibitor) (115), chondroitin sulfate (glycosaminoglycan) (116), triochastatin A (histone deacetylase inhibitor) (117) and the growth factor TGFb1 (118). However, in all these studies, drug effects were investigated exclusively *in vitro*. *In vivo* testing in preclinical animal models is required to increase the impact of these findings and investigate their IL33-MC axis specificity and potential off-target effects.

A promising example for an unbiased high-throughput approach to identify IL33-MC modulating drugs was published by Ramadan et al., They conducted a high-throughput screen of over 70,000 small molecules utilizing an AlphaLISA assay, which measures ST2-Fc fragment binding to IL33 (119). The lead compounds were then demonstrated to exhibit activity *in vitro* as well as *in vivo* in mouse models for graft vs. host disease.

### Targeting IL33/ST2

Development and characterization of inhibitors of IL33-ST2 signaling is an active field of research. Various synthetic molecules, antibodies and natural compounds either targeting the IL33-ST2 interaction directly, or inhibiting MyD88-IRAK and other downstream signaling pathways, or disrupting production of mediators are in now pre-clinical testing (74).

Targeting the IL33-ST2 interaction strategies are favored due to the knowledge gained from the naturally occurring soluble ST2 receptor isoform (sST2), a secreted “decoy receptor,” which binds IL33 and thereby sequestering the ligand from binding to membrane-bound ST2. High sST2 expression has been associated with anti-tumor responses in several cancers (120). However, the most advanced modalities targeting the IL33-ST2 interaction are antibodies, with five different anti-IL33 or anti-ST2 antibodies being tested in clinical phase 1 trials and found to be safe for use in humans (NCT02170337, NCT01928368, NCT02958436, NCT02999711, NCT03112577, NCT02345928, NCT03096795). Currently, there are multiple phase 2 trials ongoing/completed investigating the efficacy of IL33-ST2 inhibition against various allergic and inflammatory diseases and diabetic kidney disease (Table 1A).

**Table 1A:** Clinical trials utilizing antibodies targeting IL33/ST2.

**Antibody**	**Company**	**Clinical trial**	**Phase**	**Indication**	**Status/Results**
MSTT1041A,	Genentech/	NCT02918019	2b	Uncontr. severe asthma	Completed
AMG 282, RG6149	Amgen	NCT03747575	2	Atopic dermatitis	Active, not recruiting
(anti-ST2)					
REGN3500,	Sanofi/Regeneron	NCT03387852	2	asthma	Completed, met 1st & 2nd endpoint
SAR440340		NCT03546907	2	COPD^*^	Recruitment completed
(anti-IL33)		NCT03736967	2	Atopic dermatitis	Recruiting
		NCT03738423			
GSK3772847,	GSK/	NCT03207243	2a	Severe asthma	Recruitment completed
CNTO 7160 (anti-ST2)	J&J	NCT03393806	2	Asthma with AFAD^*^	Active, not recruiting
ANB020,	Anaptysbio	NCT02920021	2	Peanut allergy	Completed (121)
Etokimab		NCT03469934	2	Eosinophilic asthma	Recruitment completed
(anti-IL33)		NCT03533751	2	Atopic dermatitis	Completed (122)
		NCT03614923	2	Chron. Rhinosinusitis with NP^*^	Recruiting
MEDI3506	AstraZeneca	NCT04170543	2a	Diabetic kidney disease	Recruiting
(anti-IL33)		NCT04212169	2	Atopic dermatitis	Recruiting

* COPD, chronic obstructive pulmonary disease;

* AFAD, allergic fungal airway disease;

* *NP, Nasal Polyps*.

To date, no clinical trials have been conducted in cancer patients. Indeed, only a limited number of studies have used IL33-ST2 neutralizing antibodies in preclinical tumor models *in vivo* (Table 1B). Strikingly, all these studies demonstrated anti-tumor effects of anti-IL33 and anti-ST2 antibody treatments. However, as a cautionary tale, multiple studies demonstrate anti-tumor effects upon administration of recombinant IL33 (34, 41).

**Table 1B:** Studies utilizing antibodies IL33/ST2 in tumor models in mice.

**Reference**	**Antibody**	**Cancer model**	**Result**	**MCs analyzed**
Guabiraba et al. (123)	Anti-IL33, anti-mouse, clone 396118, MAB3626, R&D	CT26 colon cancer cell line subcutaneous	aIL33+Irinotecan -> anti-tumor effect	No
Nakagawa et al. (105)	Anti-IL33, R&D	KTC-K19CreERT extrahepatic cholangiocarcinoma mice	Anti-tumor effect	No
Wu et al. (124)	Anti-IL33, anti-human, MAB36254, R&D	Renal cancer cell lines 786O and OSRC2 subcutaneous in nude BalbC	Anti-tumor effect	No
	Anti-ST2, anti-human, Clone MAB523, R&D		Anti-tumor effect	No
Zhou et al. (125)	Rabbit anti-mouse, R&D	CT26 colon cancer cell line subcutaneous	Anti-tumor effect	No
	Rabbit anti-mouse, R&D		Anti-tumor effect	No
Kim et al. (126)	Anti-ST2, anti-mouse, clone 245707, MAB10041, R&D	KrasG12DxCCSP-Cre lung cancer model	Anti-tumor effect	No
Lin et al. (127)	Anti-ST2, monoclonal anti-human, R&D	Ln229 glioma cell line subcutaneous in NSG mice	Anti-tumor effect	NSG are MC-def.
Maywald et al. (70)	Anti-ST2, mu-IgG1-FC–anti-muST2, Amgen	ApcMin intestinal cancer model	Anti-tumor effect	Yes, MC number + activation decreased in IL33KO/anti-St2 treated tumors
Kudo-Saito et al. (128)	Anti-IL33, anti-mouse, R&D	B16F10 melanoma subcutaneous and intravenous	Anti-tumor	Yes, MC increased in BM metastasis

### Targeting MCs

A plethora of strategies to target MC receptors, intracellular signaling components and MC-derived mediators have been tested, with some now being used in the clinic. Traditionally, agents targeting MCs were studied and applied in allergies and related disorders (129, 130). Accordingly, mast cell stabilizers, drugs like Cromolyn sodium, Nedocromil, and Lodoxamide, which block MC degranulation are utilized for indications like asthma and other allergic diseases (130).

A number of tyrosine kinase inhibitors including Nilotinib, Sunitinib, Dasatinib, Imatinib, and Masitinib are in clinical trials or in clinical practice as anti-cancer drugs (130). All these small molecule inhibitors have high affinity for the tyrosine kinase receptor KIT, in addition to other tyrosine kinases. KIT is a key molecule for MC development, proliferation, survival and function and inhibition of KIT reduces MC numbers and inhibits their function. For example, Imatinib was shown to reduce asthma symptoms in a MC-dependent manner (131), yet the impact of these TK inhibitors on MCs and their contribution to the anti-tumor effect has not been investigated systematically. In the first instance, it would be important to establish whether tumors with high MC numbers respond better to anti-KIT tyrosine kinase inhibitors.

The field of targeting IL33-ST2 signaling is quickly progressing, with neutralizing antibodies being the most promising agents. While these antibodies advance rapidly in clinical trials for various inflammatory disorders, their use as anti-cancer agents is only just beginning. More work is required to better dissect tumor-promoting from tumor suppressing roles conferred by the IL33-ST2 axis in order to predict in which tumor microenvironment inhibition of IL33-ST2 signaling or MCs will be beneficial.

## Challenges for the Field

The importance of IL33 and MCs in GI cancer has been well documented. In recent years, there has been some progress in understanding the mechanisms of how the IL33-MC axis acts in GI cancers. While there is an increasing interest in targeting this signaling node in various diseases, the few drug candidates currently undergoing clinical testing have not been utilized in cancer trials yet. This is due to the dichotomous actions of IL33 and MCs in cancer. Below we dicuss some of the aspects of IL33 and MC biology which need to be addressed in order to advance the field toward harnessing IL33/MCs targeting as a novel treatment option for GI cancers.

### Diversity of Cell Types Responding to IL33

While there is now ample evidence that the IL33-MC axis is important for many cancers, the multitude of cell types in the TME able to respond to IL33 and mediated either pro- or anti-tumorigenic effects presents a formidable challenge for predicting the outcome of anti-IL33/anti-ST2 therapies. We propose that a detailed investigation of the spatial distribution of IL33-expressing cells and ST2-presenting responder cells in combination with full immunophenotyping of tumors will help addressing these issues. Since oxidation of IL33 in the extracellular space occurs rapidly and drastically reduces its ability to bind ST2 and trigger downstream signaling activation (132), we speculate that only the ST2-expressing cells in close spatial proximity of IL33-rpoducing cells will respond to IL33. Novel technologies like multiplex immunofluorescence microscopy, will allow spatial identification of cell types expressing IL33 and ST2, enabling prediction of responder cell types. Because Tregs and ILC2s are also constitutively expressing ST2, these cell types should be the included in studies attempting to predict anti-tumor effects of IL33-ST2 inhibition.

Also, further research is required to better understand the temporal dimension of IL33 secretion and the cell types responding during early vs. late stages of tumorigenesis. Indeed some studies suggest that IL33 expression is decreased in more advanced disease (97, 98) while serum levels of IL33 increased in patient with advanced gastric cancer (133). Tissue resident ST2-expressing cells, like MCs and ILC2s are the dominant IL33 responders during the early stages of tumor development. However, it is not known whether these cells can lose their responsiveness to IL33 in the changing tumor microenvironment, for example, by downregulating expression of ST2, nor has it has been investigated whether the dominant IL33 responses shift with increasing tumor size and progression of disease toward ST2-positive cells newly recruited into the tumors. Nevertheless, there is significant evidence of the role of MCs and IL33 in late stage cancers, particular in the context of tissue remodeling, epithelial to mesenchymal transition and invasion (104, 128, 134, 135).

### MC Heterogeneity

Many effects of IL33 are mediated through MC activation. However, the true extent of MC heterogeneity within the TME is not well understood. Only a few whole transcriptome studies are published, all of them were performed on bulk MCs isolated from healthy mice or humans. As part of the FANTOM5 project, Motakis et al. (136) elucidated the transcriptome of human skin MCs and compared against *ex vivo* cultured MCs. They found MC-specific gene signatures distinguishing the skin MCs from various other cell types, and discovered significant changes in gene expression profiles suggesting significant de-or trans-differentiation associated with *in vitro* propagation of MCs cultured (136). This warrants careful interpretation of findings obtained from *in vitro* studies. Transcriptional profiling of MCs from various tissues against other major immune cell lineages, revealed not only distinct differences between the various cell types but also considerable transcriptional heterogeneity between MCs recovered from different tissues (137). Indeed, a recent review suggested to replace the currently used system of histological classification of MCs with a system based on MC protease expression to more accurately reflect the tissue-specific versatility of MCs (138). Single cell sequencing studies of cancer-associated MCs are required to elucidate the true extent of mast cell heterogeneity to better understand the various biological consequences of mast cell activation in the cancer setting.

### Diversity of Mast Cell Activation Signals

Following on from the initial study by Schmitz et al., the ability of IL33 to activate MCs has been studied extensively (2, 139). However, MCs are key sentinel cells that express many receptors on their surface (46, 140), resulting in a multitude of environmental factors able to trigger their activation.

Allergen IgE-mediated activation of MCs was the first to be identified and is well characterized in the context of allergic pathologies, yet many other factors can activate MCs in an IgE-independent manner (52, 139).

Numerous studies have shown that IL33-elicited responses in MCs differ from IgE stimulation and that IL33-mediated responses in MCs are modified, and often potentiated, when secondary stimuli like IgE, substance P or IL3 are present (112, 141–144). Further research is required to uncover other MC-activating factors present in the tumor microenvironment and how they impact IL33 signaling and MC activation.

## Conclusions

Diverse functions for both IL33 and mast cells were uncovered in the context of cancer initiation and progression. However, only by focusing on the IL33/MC axis, rather than studying these key regulators of immunity separately, and by utilizing novel technologies, will the full potential of targeting IL33 signaling and MC activation be discovered and exploited for anti-cancer therapies.

## Author Contributions

MFE, MB, and ME: conception, design, writing, reviewing, and editing of the manuscript. All authors: contributed to the article and approved the submitted version.

## Conflict of Interest

The authors declare that the research was conducted in the absence of any commercial or financial relationships that could be construed as a potential conflict of interest.
